# Comparison of Machine Learning Models Including Preoperative, Intraoperative, and Postoperative Data and Mortality After Cardiac Surgery

**DOI:** 10.1001/jamanetworkopen.2022.37970

**Published:** 2022-10-26

**Authors:** José Castela Forte, Galiya Yeshmagambetova, Maureen L. van der Grinten, Thomas W. L. Scheeren, Maarten W. N. Nijsten, Massimo A. Mariani, Robert H. Henning, Anne H. Epema

**Affiliations:** 1 Department of Clinical Pharmacy and Pharmacology, University Medical Center Groningen, University of Groningen, the Netherlands; 2 Department of Anesthesiology, University Medical Center Groningen, University of Groningen, the Netherlands; 3 Bernoulli Institute for Mathematics, Computer Science and Artificial Intelligence, University of Groningen, the Netherlands; 4 Department of Critical Care, University Medical Center Groningen, University of Groningen, the Netherlands; 5 Department of Cardiothoracic Surgery, University Medical Center Groningen, University of Groningen, the Netherlands

## Abstract

**Importance:**

A variety of perioperative risk factors are associated with postoperative mortality risk. However, the relative contribution of routinely collected intraoperative clinical parameters to short-term and long-term mortality remains understudied.

**Objective:**

To examine the performance of multiple machine learning models with data from different perioperative periods to predict 30-day, 1-year, and 5-year mortality and investigate factors that contribute to these predictions.

**Design, Setting, and Participants:**

In this prognostic study using prospectively collected data, risk prediction models were developed for short-term and long-term mortality after cardiac surgery. Included participants were adult patients undergoing a first-time valve operation, coronary artery bypass grafting, or a combination of both between 1997 and 2017 in a single center, the University Medical Centre Groningen in the Netherlands. Mortality data were obtained in November 2017. Data analysis took place between February 2020 and August 2021.

**Exposure:**

Cardiac surgery.

**Main Outcomes and Measures:**

Postoperative mortality rates at 30 days, 1 year, and 5 years were the primary outcomes. The area under the receiver operating characteristic curve (AUROC) was used to assess discrimination. The contribution of all preoperative, intraoperative hemodynamic and temperature, and postoperative factors to mortality was investigated using Shapley additive explanations (SHAP) values.

**Results:**

Data from 9415 patients who underwent cardiac surgery (median [IQR] age, 68 [60-74] years; 2554 [27.1%] women) were included. Overall mortality rates at 30 days, 1 year, and 5 years were 268 patients (2.8%), 420 patients (4.5%), and 612 patients (6.5%), respectively. Models including preoperative, intraoperative, and postoperative data achieved AUROC values of 0.82 (95% CI, 0.78-0.86), 0.81 (95% CI, 0.77-0.85), and 0.80 (95% CI, 0.75-0.84) for 30-day, 1-year, and 5-year mortality, respectively. Models including only postoperative data performed similarly (30 days: 0.78 [95% CI, 0.73-0.82]; 1 year: 0.79 [95% CI, 0.74-0.83]; 5 years: 0.77 [95% CI, 0.73-0.82]). However, models based on all perioperative data provided less clinically usable predictions, with lower detection rates; for example, postoperative models identified a high-risk group with a 2.8-fold increase in risk for 5-year mortality (4.1 [95% CI, 3.3-5.1]) vs an increase of 11.3 (95% CI, 6.8-18.7) for the high-risk group identified by the full perioperative model. Postoperative markers associated with metabolic dysfunction and decreased kidney function were the main factors contributing to mortality risk.

**Conclusions and Relevance:**

This study found that the addition of continuous intraoperative hemodynamic and temperature data to postoperative data was not associated with improved machine learning–based identification of patients at increased risk of short-term and long-term mortality after cardiac operations.

## Key Points

**Question:**

Is adding continuous intraoperative data to routinely collected perioperative data associated with improved machine learning–based mortality predictions after cardiac bypass and valve operations?

**Findings:**

In this prognostic study of 9415 patients who underwent first-time cardiac surgery, machine learning–based prediction of mortality using preoperative, intraoperative, and postoperative data was not associated with improved performance or clinical utility of models based on postoperative data only. Postoperative markers associated with metabolic dysfunction and decreased kidney function were the main factors contributing to mortality risk.

**Meaning:**

These findings suggest that there is unclear value in adding continuous, high-dimensional intraoperative hemodynamic and temperature data to machine learning models that use relatively easy-to-obtain, limited postoperative data to predict mortality in patients undergoing cardiac surgery.

## Teaser

This prognostic study compares machine learning models that use preoperative, intraoperative, and postoperative data to predict mortality after cardiac surgery.

## Introduction

Postoperative mortality risk is associated with several preoperative, intraoperative, and postoperative factors, and age, comorbidities, and decreased preoperative kidney function are known factors associated with increased risk.^1,2,3,4,5,6^ Current preoperative risk stratification methods, such as the European System for Cardiac Operative Risk Evaluation (EuroSCORE) II^7^ and American Society of Thoracic Surgeons score,^8,9^ therefore use these preoperative variables to predict short-term risk. Among postoperative factors, acute kidney injury (AKI) is 1 of the best characterized factors associated with risk for mortality in coronary artery bypass grafting (CABG) and valve operations.^1,10^ However, the usefulness of routinely collected and more complex intraoperative and postoperative clinical parameters other than kidney function to predict short-term and especially long-term mortality remains understudied.

Machine learning (ML) models can process and use large amounts of data collected before, during, and after surgery. Indeed, an increasing number of ML models have been published that use preoperative data and predict mortality and complications after cardiac surgery more accurately than traditional risk scores.^11,12,13,14^ Similarly, we previously found that routinely collected postoperative data was associated with improved prediction of long-term mortality compared with traditional statistical analyses.^5,15^ However, ML models are most promising due to their ability to dynamically take in and produce predictions based on data across different phases of the entire perioperative period (preoperative, intraoperative, and postoperative), all of which differ in data type and density. The intraoperative phase, in particular, produces large quantities of continuous data, such as time series of hemodynamic, temperature, and cardiopulmonary bypass (CPB) perfusion measurements, which require different modeling than other perioperative data. A 2021 study^16^ and a 2019 study^17^ found that using interventional and continuous intraoperative data or aggregated intraoperative data were associated with improved 30-day postoperative mortality predictions after cardiac operations. However, it is unclear whether combining preoperative, intraoperative, and postoperative data is associated with improved predictions of short-term and long-term mortality.

In this study, we developed an ML algorithm combining a long short-term memory (LSTM) neural network modeling 12 routinely collected intraoperative hemodynamic and temperature variables. Additionally, we developed a gradient-boosted classifier to predict short-term and long-term mortality from a large prospectively collected single-center registry of perioperative data of patients who underwent cardiac surgery. We analyzed the prediction outcomes and clinical utility associated with the addition of intraoperative data to models predicting 30-day, 1-year, and 5-year mortality and investigated factors contributing to these predictions with feature importance analysis.

## Methods

The Medical Ethical Committee of the University Medical Centre Groningen granted this prognostic study a waiver from review because according to Dutch Law, studies in which data are collected as part of standard care without the need for additional measurements do not require ethical review board approval. Consent was obtained before data collection from all patients. This study followed the Transparent Reporting of a Multivariable Prediction Model for Individual Prognosis or Diagnosis (TRIPOD) reporting guideline for prognostic studies.

### Data Source

The electronic Cardiothoracic Anesthesiology Registry (CAROLA) comprises perioperative data of a prospective cohort of patients from our tertiary center undergoing cardiac operations between 1997 and 2017 in the University Medical Centre Groningen in the Netherlands. Mortality data were obtained from the Dutch Municipal Personal Records Database, which comprises actual and reliable data of all citizens within the Netherlands obtained in November 2017.

### Patient Population and Outcome

Data comprised 13 944 patients, of whom 9415 underwent a first-time CPB-assisted elective valve operation or CABG or a combination of both. The primary outcomes were 30-day, 1-year, and 5-year mortality rates. The secondary outcome was the contribution of variables to the outcome determined using Shapley additive explanations (SHAP) analysis.

### Data Selection and Preprocessing

The preoperative data set consisted of routinely collected patient characteristics and laboratory variables recorded before surgery available in CAROLA. Except for a clear demarcation in EuroSCORE between patients included in the last 10 to 12 years and patients included earlier (due to the use of EuroSCORE II and I), there have not been noteworthy changes in data collected. Intraoperative data included continuous monitoring time series of hemodynamic, temperature, and CPB data aggregated per minute. Postoperative data consisted of laboratory values recorded at least once daily during the postsurgery period.

To account for a variable pattern of missing data preoperatively, we separated preoperative laboratory variables into 2 categories (ie, measured less than 24 hours or more than 24 hours before surgery) and aggregated them by taking the mean. Multivariate feature imputation was used for missing preoperative values, with custom thresholds for each variable defined.^18^ This resulted in a set of 20 static preoperative variables.

For intraoperative data, 12 continuous monitoring variables were included. For all variables, acceptable thresholds were defined to filter out artifacts, and a rolling mean was calculated. Interpolation and backward and forward propagation were used for missing values. Given that registration of each intraoperative variable starts at different time points, we defined 3 distinct intraoperative periods (before, during, and after CPB). Nasopharyngeal, rectal, and skin temperature data were consistently recorded only during bypass, and some variables were considered only before and after but not during perfusion because of signal perturbations caused by the heart-lung machine. Patients with an irregular duration of surgery due to registration artifacts (ie, an operation time >1440 minutes) were excluded.

Finally, postoperative data were split into 6 time periods (first 6 hours and hours 6-12, 12-24, 24-48, 48-72, and 72-96). If more than 1 laboratory value for a variable was present per time period, the mean of those measurements was taken. The analysis included 91 postoperative variables.

### Model Development

The predictive algorithm included a recurrent neural network with an LSTM architecture. The neural network processed preoperative and intraoperative data and then output its individual hidden states to a gradient-boosted algorithm that combined postoperative with preoperative and intraoperative states.

Recurrent neural networks are a class of neural networks with feedback loops that are increasingly used for time-series processing in anesthesiology, surgery, and critical care.^17,19,20,21^ In particular, LSTM recurrent neural networks are optimal to model sequential inputs owing to their feedback loops that create a memory of previous inputs and store these in a hidden state, enabling networks to learn long-term dependencies.^22,23,24^ The flow of information is regulated by input gates, which control information from the input; forget gates, which determine the weight for specific data in the feedback loop; and output gates, which determine what is sent to other units. Gradient boosting is a widely used technique in ML regression and classification in which the loss function is optimized by sequentially combining weak learners (usually decision trees) to generate an additive, gradient descent model with increasingly better performance.^25^ The extreme gradient boosting machine (XGBoost) algorithm is a scalable decision tree–based boosting algorithm that increasingly weighs difficult-to-predict events using *k*-fold cross-validation. We previously showed that this algorithm performs well in prediction of long-term mortality after CABG.^5,26^

**Figure 1.**
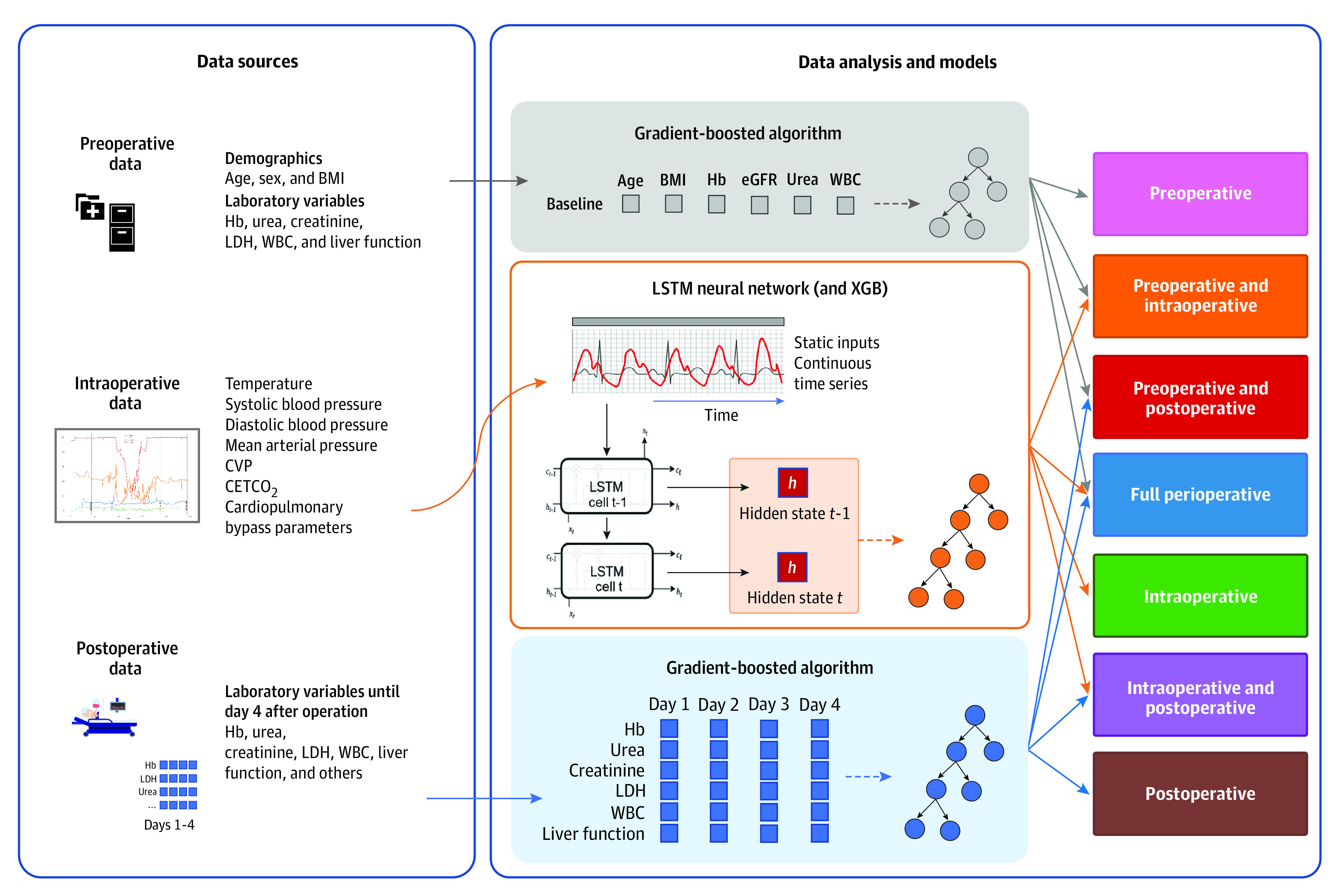
Schematic Overview of Analysis Raw data of patients undergoing 1 of 3 cardiac operations were aggregated from the Cardiothoracic Anesthesiology Registry database. Data from 3 perioperative periods were then preprocessed and fed into the long short-term memory (LSTM) and gradient-boosted algorithm; 7 models were generated to predict short-term and long-term mortality and classify patients as low or high risk. BMI indicates body mass index (calculated as weight in kilograms divided by height in meters squared); CETCO_2_, end-tidal carbon dioxide; CVP, central venous pressure; Hb, hemoglobin; eGFR, estimated glomerular filtration rate; LDH, lactate dehydrogenase; WBC, white blood cell; XGB, extreme gradient boosting.

To conduct this analysis, we built the pipeline shown in Figure 1. To pass preoperative data to the LSTM for full perioperative models, static preoperative features were first processed with principal components analysis to decrease the dimensionality of the data.^27^ These data were then merged with intraoperative data and passed together to the first hidden layer of the LSTM. This was done to ensure that static data did not pollute intraoperative sequences. Next, to obtain representations of the intraoperative condition of a patient at different time points, the LSTM was trained to predict the hidden state in the subsequent LSTM cell. These hidden-state representations were combined with postoperative sequential data and passed into an XGBoost classifier, which was trained with 10-fold cross-validation, to produce final predictions. This step, similar to PCA for preoperative data, was required due to the difference in dimensionality between high-frequency intraoperative and low-frequency preoperative and postoperative data. For models including only preoperative or postoperative data, or a combination thereof, only XGBoost was used.

### Explainability of Predictions With Variable Importance Analysis

Identifying relevant factors and how they contribute to a prediction is an important step for evaluating targeted interventions in a clinical setting.^28^ To test how individual factors in this analysis contributed to mortality or survival predictions, the SHAP algorithm was applied to the gradient-boosted model. Shapley values are widely used metrics in cooperative game theory, and in the context of ML, they help evaluate the contribution of any particular feature to the difference between actual and mean predictions.^28,29^ Feature contribution is calculated as the change in the expected value of the model’s output when a given feature is observed vs when it is unknown.^28^ These values can be quantified and graphically represented. In this study, variables graphically represented in red indicated contribution toward mortality, while variables represented in blue indicated contribution to survival.

### Statistical Analysis

Model hyperparameters were tuned using a grid search during training with 80% of the data. The remaining 20% of the test set was used for validation and to compute performance results (eTable 1 in the Supplement).

We assessed the performance of 7 models generated in this study: those using data from (1) preoperative, (2) intraoperative, (3) postoperative, (4) preoperative and intraoperative, (5) intraoperative and postoperative, (6) preoperative and postoperative, and (7) preoperative, intraoperative, and postoperative (ie, full perioperative) periods. Performance was assessed using area under the receiver operator characteristic curve (AUROC), with sensitivity, specificity, and positive and negative predictive values also reported.^30^ Differences in performance between models were assessed with the DeLong nonparametric test for the difference in AUROC.^31^ To assess the potential clinical relevance of predictions, we plotted predictiveness curves to show the distributions of risk scores for mortality at each follow-up time. Patients were then classified as high or low risk for mortality based on the risk distribution derived from predictiveness curves.^32^ Relative risk was calculated as the ratio of the absolute risk of mortality between 2 groups. Actual observed mortality was plotted against the predicted probability of mortality in the test set to explore the quality of the calibration, which was assessed visually.^33,34^ All metrics are reported with 95% CIs, and 2-tailed tests were considered statistically significant at *P* < .05. All analyses were conducted using the scikit-learn module version 0.22.1 in the Python programming language version 3.9.5 (Python Software Foundation) and occurred between February 2020 and August 2021.^35^

## Results

Among 9415 patients (median [IQR] age, 68 [60-74] years; 2554 [27.1%] women) included in the analysis, 5547 patients underwent CABG (58.9%), 2535 patients underwent solitary valve surgery (26.9%), and 1333 patients underwent combined valve and coronary surgery (14.2%). verall mortality rates at 30 days, 1 year, and 5 years were 268 patients (2.8%), 420 patients (4.5%), and 612 patients (6.5%), respectively. Valve surgery had the highest mortality rates, followed by combined and CABG surgery (eFigure 1 in the Supplement). Baseline characteristics of patients stratified by survival status are presented in Table 1 and eTable 2 in the Supplement.

**Table 1.** Patient Characteristics

Characteristic^a^	Patients, No. (%) (N = 9415)		*P* value
	Survivors (n = 8611)	Nonsurvivors (n = 804)	
**Demographic characteristic**			
BMI, mean (95% CI)	20.4 (20.3-20.5)	20.6 (20.5-20.8)	<.001
Age, median (IQR), y	67.0 (74.0-15.0)	71.0 (77.0-12.0)	<.001
Sex			
Women	2554 (27.1)	270 (2.9)	.02
Men	6057 (64.3)	534 (5.7)	
Operation			
CABG	5126 (59.5)	421 (52.4)	<.001
Valve	2310 (26.8)	225 (28.0)	<.001
Combined	1175 (13.6)	158 (19.7)	<.001
**Laboratory value, mean (95% CI)**			
eCCR, mL/min/1.73 m^2^			
Preoperative	76.3 (72.8-79.8)	83.2 (72.8-93.6)	.11
Postoperative	92.3 (90.2-94.0)	85.0 (80.1-89.8)	<.001
Creatinine, mg/dL			
Preoperative	1.1 (1.09-1.11)	1.43 (1.35-1.52)	<.001
Postoperative	1.02 (1.01-1.03)	1.49 (1.40-1.56)	<.001
Urea nitrogen, mg/dL			
Preoperative	22.1 (21.6-22.4)	27.2 (25.6- 28.3)	<.001
Postoperative	35.6 (34.5-36.7)	42.3(39.5-45.1)	<.001
LDH, U/L			
Preoperative	233.3 (230.9-235.7)	287.7 (268.3-307.1)	<.001
Postoperative	353.4 (349.4-357.4)	623.6 (580.1-667.1)	<.001
Postoperative blood glucose, mg/dL	158.6 (158.6-160.4)	169.4 (165.8-171.2)	<.001
Hb, g/dL			
Preoperative	15.3 (15.0-15.5)	16.4 (14.5-18.5)	.03
Postoperative	10.2 (10.0-10.3)	9.7 (9.3-10.0)	<.001
Leukocytes, /μL			
Preoperative	8500 (8400-8700)	10 000 (8000-12 000)	<.001
Postoperative	13 800 (13 700-13 900)	15 200 (14 300-16 000)	<.001
Platelet count, ×/μL			
Preoperative	237.4 (235.8-238.9)	233.6 (227.5-239.8)	.17
Postoperative	161.3 (160.2-162.5)	144.5 (140.1-148.9)	<.001
ALT, U/L			
Preoperative	36.9 (35.9-37.9)	34.9 (32.3-37.5)	.22
Postoperative	40.6 (38.4-42.9)	103.4 (85.3-121.5)	<.001
AST, U/L			
Preoperative	34.3 (33.3-35.3)	41.5 (35.7-47.2)	<.001
Postoperative	56.4 (54.5-58.2)	157.5 (132.9-182.0)	<.001
Postoperative ESR, mm/h	19.7 (19.3-20.0)	27.7 (26.1-29.3)	<.001
ESR <24 h before surgery, mm/h	19.5 (19.1-19.8)	27.8 (26.2-29.5)	<.001
Postoperative WBC count, ×/μL			
Neutrophils	11 800 (11 800-11 900)	12 500 (12 100-12 800)	<.001
Monocytes	3600 (3600-3700)	3700 (3600-3900)	.38
Lymphocytes	1400 (1300-1400)	1900 (1600-2100)	.004
**Intraoperative variable, mean (95% CI)**			
HR during perfusion, bpm	63.2 (62.5-64.0)	67.6 (65.3-69.9)	.001
SBP during perfusion, mmHg	70.6 (70.4-70.9)	69.5 (68.5-70.5)	.020
DBP during perfusion, mmHg	58.3 (58.1-58.6)	56.4 (55.5-57.3)	<.001
CVP during perfusion, mmHg	6.8 (6.7-6.9)	7.6 (6.7-8.5)	.002
Duration of perfusion, min	127.4 (126.2-128.6)	152.7 (146.9-158.5)	<.001
Minimum body temperature, °C	28.4 (28.3-28.5)	27.5 (27.2-27.8)	<.001

Abbreviations: ALT, alanine aminotransferase; AST, aspartate aminotransferase; BMI, body mass index (calculated as weight in kilograms divided by height in meters squared); bpm, beats per minute; CABG, coronary artery bypass grafting; CVP, central venous pressure; DBP, diastolic blood pressure; eCCR, estimated creatinine clearance; ESR, erythrocyte sedimentation rate; Hb, hemoglobin; HR, heart rate; LDH, lactate dehydrogenase; SBP, systolic blood pressure; WBC, white blood cell.

SI conversion factors: To convert ALT, AST, and LDH to microkatals per liter, multiply by 0.0167; eCCR to milliliters per second per meters squared, multiply by 0.0167; ESR to mm/h, multiply by 1.0; creatinine to micromoles per liter, multiply by 88.4; glucose to micromoles per liter, multiply by 0.0555; Hb to grams per liter, multiply by 10.0; platelet count to ×10^9^/L, multiply by 1.0; urea nitrogen to millimoles per liter, multiply by 0.357; WBC counts to ×10^9^/L, multiply by 0.001.

^a^ Categorical variables are represented as number and percentage. Nonparametric variables are reported as median (IQR) and compared using Wilcoxon signed-rank test. Parametric continuous variables are presented as mean (95% CI) and compared using student *t* test.

### Model Performance and Calibration

**Figure 2.**
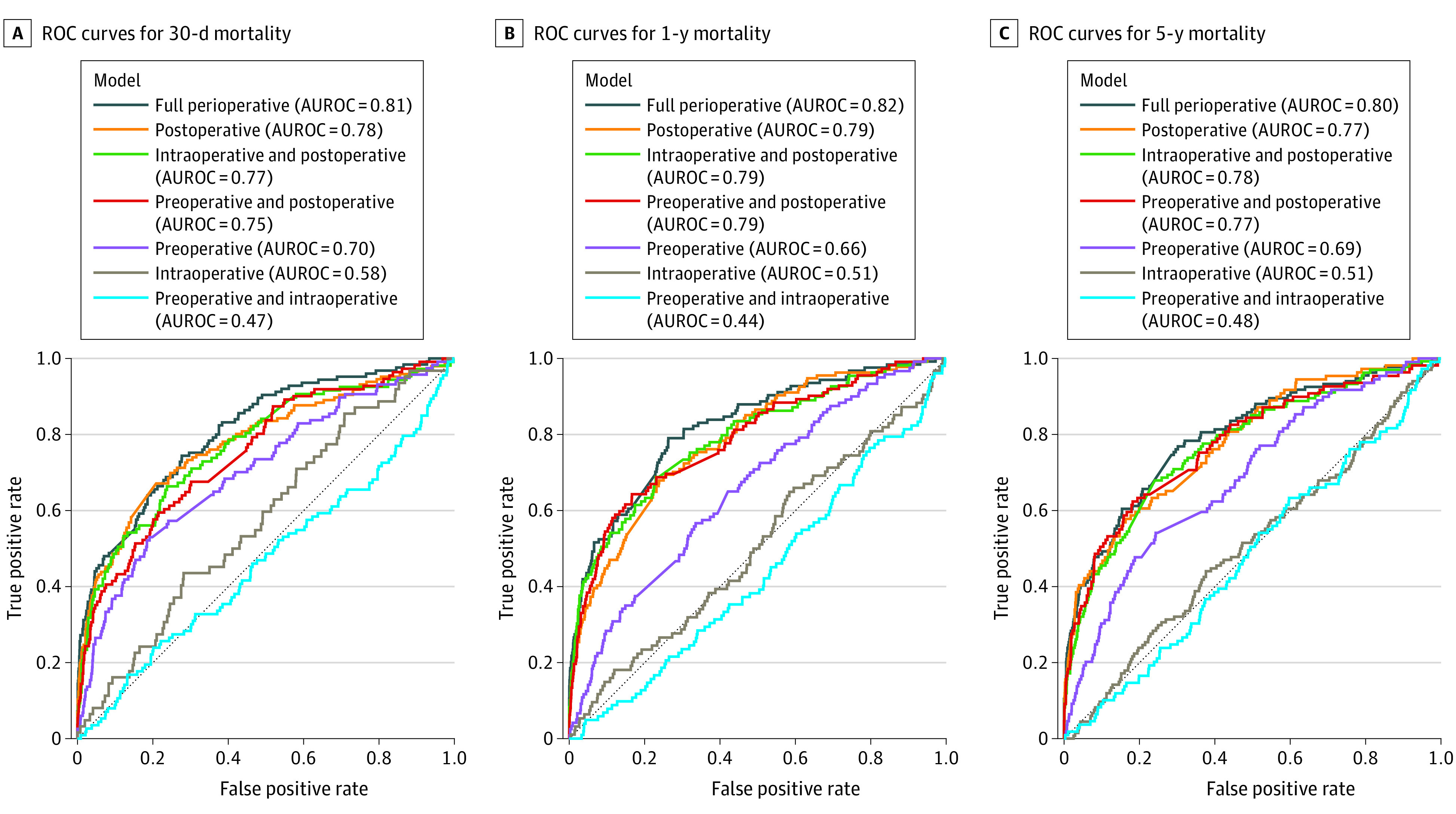
Model Performance by Mortality Follow-up Point Receiver operating characteristic (ROC) curves and area under the ROC curve (AUROC) values are given.

Model performance is reported in Figure 2. Models using exclusively preoperative data achieved AUROC values of 0.70 (95% CI, 0.61-0.71), 0.66 (95% CI, 0.61-0.71), and 0.69 (95% CI, 0.64-0.74) for 30-day, 1-year, and 5-year mortality, respectively. Models including preoperative and intraoperative data or intraoperative data only performed poorly, with AUROC values among the 3 mortality outcomes ranging from 0.44 (95% CI, 95% CI, 0.37-0.49) for 1-year mortality using preoperative and intraoperative data to 0.58 (95% CI, 0.51-0.64) for 30-day mortality using interoperative data (eTables 3, 4, and 5 in the Supplement). Other models combining preoperative or intraoperative and postoperative data performed better, achieving AUROC values ranging from 0.75 (95% CI, 0.70-0.80) for 30-day mortality using preoperative and postoperative data to, for example, 0.79 (95% CI, 0.74-0.84) for 1-year mortality using intraoperative and postoperative data. AUROC values for postoperative-only models were 0.78 (95% CI 0.73-0.82), 0.79 (95% CI, 0.74-0.83), and 0.77 (95% CI, 0.73-0.82) for 30-day, 1-year, and 5-year mortality, respectively. Fully perioperative models had higher AUROC values than models using postoperative data only, although these differences were not statistically significant, with AUROC values of 0.82 (95% CI, 0.78-0.86), 0.81 (95% CI, 0.77-0.85), and 0.80 (95% CI, 0.75-0.84) for 30-day, 1-year, and 5-year mortality, respectively. In visual assessment of calibration curves, fully perioperative and postoperative models were well-calibrated (eFigures 2, 3, and 4 in the Supplement).

### Sensitivity Analysis and Risk Stratification

We used predictiveness and classification plots to assess the distribution of patients by risk probability (eFigures 5 and 6 in the Supplement). Of 1883 patients in the validation set, full perioperative models classified 375 patients (19.9%), 268 patients (14.2%), and 280 patients (14.9%) as high risk for 30-day, 1-year, and 5-year mortality, respectively. For models using postoperative data only, 254 patients (13.5%), 266 patients (13.1%), and 54 patients (2.9%) were classified as high risk for 30-day, 1-year, and 5-year mortality, respectively. These models performed similarly in identifying patients who did not survive at 30 days and at 1 year (Table 2). For 5-year mortality, the fully perioperative model achieved better stratification than the postoperative model, with a sensitivity of 50.0% (95% CI, 41.2%-58.3%) vs 18.7% (95% CI, 12.4%-25.4%), a 31.3 percentage point increase. However, the postoperative model incorrectly classified as few as 1.7% of individuals at 5 years (specificity, 98.3% [95% CI, 97.7%-99.0%]) (Table 2). The negative predictive values for all models were greater than 94%. The positive predictive values (PPVs) of the postoperative-only model were higher than those of perioperative models for 30-day mortality (28.0% [95% CI, 22.6%-33.6%] vs 19.7% [95% CI, 15.6%-23.8%]; 8.3 percentage point increase) and 5-year mortality (46.3% [95% CI, 32.8%-60.0%] vs 23.9% [95% CI, 18.7%-29.1%]; 22.4 percentage point increase), with the latter systematically classifying more individuals as high risk (Table 2).

**Table 2.** Model Performance

Model data	Patients classified, No. (%) (n = 1883)^a^		RR (95% CI)^b^	Sensitivity (95% CI), %	Specificity (95% CI), %	PPV (95% CI), %	NPV (95% CI), %
	Low risk	High risk					
30 d							
Full perioperative	1508 (80.1)	375 (19.9)	3.5 (2.9-4.1)	59.2 (50.3-67.9)	82.9 (81.2-84.6)	19.7 (15.6-23.8)	96.6 (95.6-97.5)
Postoperative only	1629 (86.5)	254 (13.5)	4.6 (3.7-5.7)	48.6 (40.9-56.4)	89.5 (88.0-90.8)	28.0 (22.6-33.6)	95.4 (94.3-96.4)
1 y							
Full perioperative	1615 (85.8)	268 (14.2)	4.7 (3.8-5.8)	54.0 (45.1-63.1)	88.6 (87.2-90.1)	25.0 (19.6-30.6)	96.5 (95.6-97.4)
Postoperative only	1617 (85.9)	266 (13.1)	4.1 (3.3-5.1)	47.0 (38.4-55.2)	88.4 (86.8-89.8)	23.7 (18.5-28.5)	95.6 (94.5-96.6)
5 y							
Ful perioperative	1603 (85.1)	280 (14.9)	4.1 (3.3-5.1)	50.0 (41.2-58.3)	87.8 (86.3-89.3)	23.9 (18.7-29.1)	95.8 (94.8-96.7)
Postoperative only	1829 (97.1)	54 (2.9)	11.3 (6.8-18.7)	18.7 (12.4-25.4)	98.3 (97.7-99.0)	46.3 (32.8-60.0)	94.0 (93.0-95.1)

Abbreviations: NPV, negative predictive value; PPV, positive predictive value; RR, relative risk.

^a^ All percentages are out of a fraction of the cohort, equal to the sum of patients at high risk or low risk.

^b^ Risk increase computed as the relative risk between low-risk and high-risk groups.

Patients classified as high risk by full perioperative models had a 3.5-fold (95% CI, 2.9-fold to 4.1-fold), 4.7-fold (95% CI, 3.8-fold to 5.8-fold), and 4.1-fold (95% CI, 3.3-fold to 5.1-fold) relative risk increase for 30-day, 1-year, and 5-year mortality, respectively. For postoperative models, being classified as high risk had a 4.6-fold (95% CI, 3.7-fold to 5.7-fold), 4.1-fold (95% CI, 3.3-fold to 5.1-fold), and 11.3-fold (95% CI, 7-18.7) risk increase for 30-day, 1-year, and 5-year mortality, respectively.

### Feature Importance

**Figure 3.**
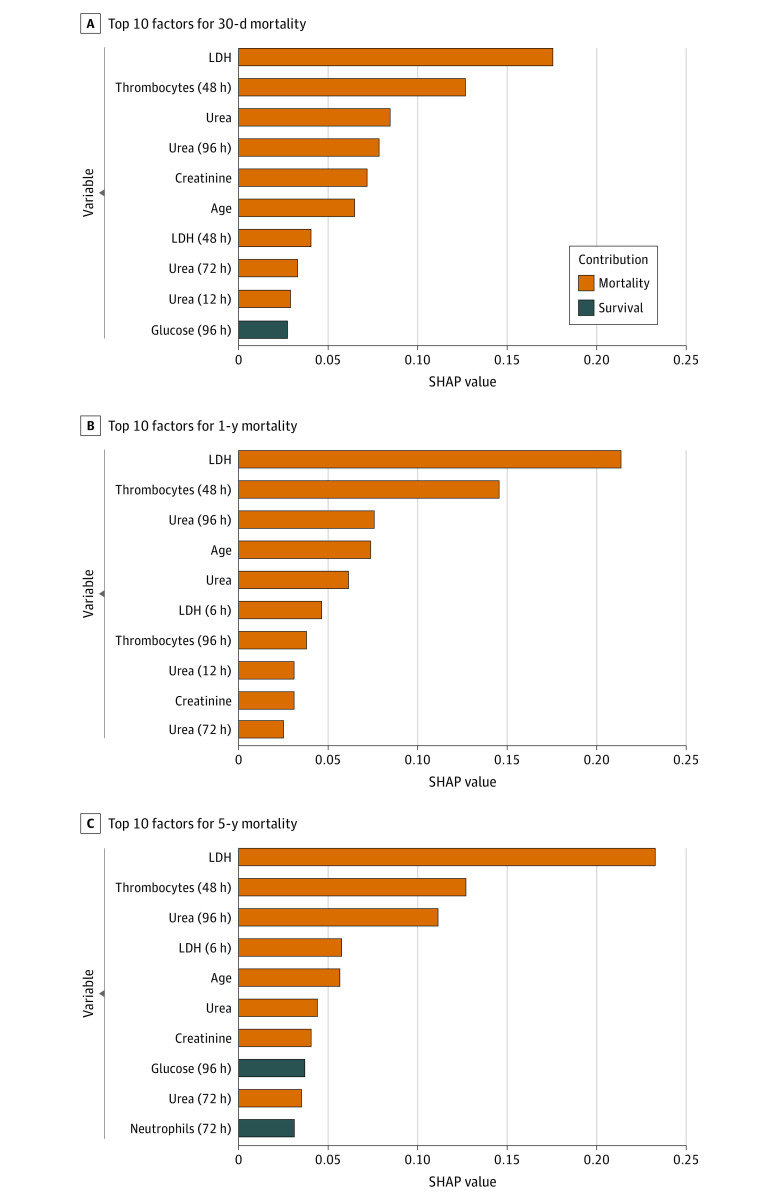
Contributions of Input Features to Mortality Predictions Positive Shapley additive explanation (SHAP) values represent the theoretical contribution of the factor to mortality. LDH indicates lactate dehydrogenase.

The contributions of variables to predictions of best-performing models by mortality outcome and type of surgery are presented in Figure 3 and eFigures 7, 8, and 9 in the Supplement. Intraoperative parameters represented by hidden states of the LSTM did not contribute to predictions in any model. At 30 days, 1 year, and 5 years, the top 10 features contributing to predictions consisted of primarily postoperative variables, particularly for markers associated with metabolic dysfunction and decreased kidney function (Figure 3). Higher mean lactate dehydrogenase (LDH) and urea levels across the first 4 postoperative days and a higher platelet count 48 hours after surgery contributed to predictions of nonsurvival at 30 days (Figure 3A). The inverse association was seen for glucose at different time points. For long-term mortality predictions, higher mean LDH and urea levels at different time points contributed to predictions of nonsurvival (Figure 3B and C).

## Discussion

In this prognostic study, we evaluated outcomes and clinical utility associated with the addition of intraoperative data to ML models predicting short-term and long-term mortality in patients undergoing cardiac surgery. Our key findings were that the addition of continuous intraoperative data to postoperative data was not associated with improved model performance or more clinically usable predictions. Additionally, postoperative markers associated with metabolic dysfunction and decreased kidney function were the main contributors to short-term and long-term mortality risk.

ML models predicting mortality after cardiac surgery remain scarce, with most studies focusing on short-term postoperative mortality. This study adds to that limited but emerging literature and is, to our knowledge, the first study to use ML to simultaneously predict short-term and long-term mortality using preoperative, intraoperative, and postoperative data. Studies from 2019 to 2021^16,17,36^ using ML algorithms have predicted 30-day mortality with AUROC values greater than 0.80 and reported that combining preoperative and intraoperative data was associated with improved mortality and AKI predictions. While our best-performing models showed a comparable discrimination, the lack of significant improvement with the addition of intraoperative data was unexpected. One possible explanation for these results lies in the nature of continuous intraoperative data. While modern intraoperative hemodynamic and temperature monitoring allows for these data to be reliably measured and stored, it also facilitates their tight regulation and optimization by anesthesiologists. Therefore, intraoperative data on events and interventions, such as blood transfusions and hemodynamic medication administration, may better reflect the actual intraoperative hemodynamic state of patients undergoing cardiac surgery. Indeed, blood transfusions and prolonged intraoperative hypotension are associated with one another and may predict postoperative outcomes.^16,37,38,39^ On the contrary, several meta-analyses and randomized clinical trials^40,41,42^ have found no clinical difference in morbidity and mortality between groups with different perioperative blood pressure targets in cardiac and noncardiac surgical populations, which further brings into question the value of modeling hemodynamic data for mortality prediction. These findings suggest that additional research will be needed to optimize intraoperative data modeling when incorporating data from the entire perioperative period and to maximize the potential clinical utility of these models.

Another challenge in optimizing the clinical utility of ML models resides in balancing true and false positives in specific clinical settings. In cardiac surgical cohorts, in which mortality is relatively low, it may be desirable for models to aim for high sensitivity and PPV. This may help ensure that patients at greater risk are identified before discharge, without excessively increasing clinical burden given that the proportion of patients at risk is inherently low. While we hypothesized that the inclusion of intraoperative date would be associated with improvements in this area, 30-day sensitivity and PPVs of full perioperative and postoperative-only models were comparable.

Unlike preoperative factors, intraoperative and early postoperative factors do not have well-established importance for mortality prediction. In a previous study,^5^ we identified high postoperative urea as the factor with the greater contribution to 5-year postoperative mortality. This could potentially be explained by changes in urea reflecting multiorgan pathology or mitochondrial dysfunction caused by the ischemia or reperfusion and systemic inflammatory response associated with CPB and surgical trauma.^43,44^ In this study, high LDH, urea, and creatinine values during the first postoperative days had large contributions to mortality for 30-day and 1-year outcomes. Like urea, LDH is associated with CPB damage, hypoxia, accelerated aerobic metabolism, and prolonged rhythm abnormalities, such as ventricular fibrillation during perfusion.^45,46,47^ Examining creatinine, a 2021 retrospective latent class analysis^48^ found as many as 12 reproducible AKI classes based on serum creatinine trajectory phenotypes in patients after CABG; of these classes, 4 had a higher risk of poor outcome.

We found a predominance of postoperative factors among the main factors contributing to mortality, in contrast to baseline factors, such as age and intraoperative parameters. These results support findings of a 2012 study^9^ in which postoperative factors, such as dialysis-dependent kidney failure or development of insulin-dependent diabetes, had substantially greater contribution to mortality predictions after 2 years compared with preoperative factors.

### Limitations

This study has several limitations. Because this was a single-center study, our findings need confirmation by external validation, ideally in a prospective, multicenter setting. In addition, we applied multivariate feature imputation for missing preoperative and postoperative values and modeled intraoperative data using custom thresholds to mitigate outcomes associated with missing data. However, it cannot be excluded that this may have caused some bias, especially for postoperative time-series data. Additionally, as discussed previously, we restricted intraoperative data to hemodynamics, temperature, and CPB and did not include interventional data, such as blood transfusion or the use of inotropes or insulin. The code used in this study is available online,^49^ and we encourage further replication and validation of the algorithm and findings of this study in other cohorts, as well as the addition of new preoperative and intraoperative data types to the analysis.

## Conclusions

In this prognostic study, we compared the performance of ML models with data from all 3 perioperative periods to predict 30-day, 1-year, and 5-year mortality after cardiac surgery and investigated factors contributing to these predictions. We found that including preoperative, intraoperative, and postoperative data was not associated with improved clinical utility of ML models for short-term and long-term predictions. Postoperative markers associated with metabolic dysfunction and decreased kidney function were the main contributors to mortality risk, although further research is required to explore physiological processes that may explain this.

## Notes

Supplement. **eTable 1.** Grid Search Hyperparameter Ranges for Long Short-Term Memory Neural Network and Extreme Gradient-Boosting Machine Algorithms **eTable 2.** Complete Demographic Characteristics and Preoperative, Intraoperative, and Postoperative Variables **eTable 3.** Area Under Receiver Operating Characteristic Curves for Models for 30-d Mortality Prediction **eTable 4.** Area Under Receiver Operating Characteristic Curves for Models for 1-y Mortality Prediction **eTable 5.** Area Under Receiver Operating Characteristic Curves for Models for 5-y Mortality Prediction **eFigure 1.** Survival Curves and Survival Statistics for Entire Cohort and by Operation Type **eFigure 2.** Calibration Plot for Model Predicting 30-d Mortality in Full Cohort **eFigure 3.** Calibration Plot for Model Predicting 1-y Mortality in Full Cohort **eFigure 4.** Calibration Plot for Model Predicting 5-y Mortality in Full Cohort **eFigure 5.** Predictiveness Plots for Models Including Data From All 3 Perioperative Periods **eFigure 6.** Predictiveness Plots for Models Including Postoperative Data Only **eFigure 7.** Contribution of Input Features to Mortality Predictions for Coronary Artery Bypass Grafting Operations **eFigure 8.** Contributions of Input Features to Mortality Predictions for Valve Operations **eFigure 9.** Contributions of Input Features on Mortality Predictions for Combined Operations
