# Supplementary material for: Comparison of Machine Learning Models Including Preoperative, Intraoperative, and Postoperative Data and Mortality After Cardiac Surgery
Source: JAMA Netw Open. 2022 Oct 26;5(10):e2237970. doi: 10.1001/jamanetworkopen.2022.37970 (PMC9606847; doi:10.1001/jamanetworkopen.2022.37970)
Supplement: Supplement. — eTable 1. Grid Search Hyperparameter Ranges for Long Short-Term Memory Neural Network and Extreme Gradient-Boosting Machine Algorithms eTable 2. Complete Demographic Characteristics and Preoperative, Intraoperative, and Postoperative Variables eTable 3. Area Under Receiver Operating Characteristic Curves for Models for 30-d Mortality Prediction eTable 4. Area Under Receiver Operating Characteristic Curves for Models for 1-y Mortality Prediction eTable 5. Area Under Receiver Operating Characteristic Curves for Models for 5-y Mortality Prediction eFigure 1. Survival Curves and Survival Statistics for Entire Cohort and by Operation Type eFigure 2. Calibration Plot for Model Predicting 30-d Mortality in Full Cohort eFigure 3. Calibration Plot for Model Predicting 1-y Mortality in Full Cohort eFigure 4. Calibration Plot for Model Predicting 5-y Mortality in Full Cohort eFigure 5. Predictiveness Plots for Models Including Data From All 3 Perioperative Periods eFigure 6. Predictiveness Plots for Models Including Postoperative Data Only eFigure 7. Contribution of Input Features to Mortality Predictions for Coronary Artery Bypass Grafting Operations eFigure 8. Contributions of Input Features to Mortality Predictions for Valve Operations eFigure 9. Contributions of Input Features on Mortality Predictions for Combined Operations [file jamanetwopen-e2237970-s001.pdf]

Supplemental Online Content

Castela Forte J, Yeshmagambetova G, van der Grinten ML, et al. Comparison of machine learning models including preoperative, intraoperative, and postoperative data and mortality after cardiac surgery. *JAMA Netw Open*. 2022;5(10):e2237970. doi:10.1001/jamanetworkopen.2022.37970

eTable 1. Grid Search Hyperparameter Ranges for Long Short-Term Memory Neural Network and Extreme Gradient-Boosting Machine Algorithms
eTable 2. Complete Demographic Characteristics and Preoperative, Intraoperative, and Postoperative Variables
eTable 3. Area Under Receiver Operating Characteristic Curves for Models for 30-d Mortality Prediction
eTable 4. Area Under Receiver Operating Characteristic Curves for Models for 1-y Mortality Prediction
eTable 5. Area Under Receiver Operating Characteristic Curves for Models for 5-y Mortality Prediction
eFigure 1. Survival Curves and Survival Statistics for Entire Cohort and by Operation Type
eFigure 2. Calibration Plot for Model Predicting 30-d Mortality in Full Cohort
eFigure 3. Calibration Plot for Model Predicting 1-y Mortality in Full Cohort
eFigure 4. Calibration Plot for Model Predicting 5-y Mortality in Full Cohort
eFigure 5. Predictiveness Plots for Models Including Data From All 3 Perioperative Periods
eFigure 6. Predictiveness Plots for Models Including Postoperative Data Only
eFigure 7. Contribution of Input Features to Mortality Predictions for Coronary Artery Bypass Grafting Operations
eFigure 8. Contributions of Input Features to Mortality Predictions for Valve Operations
eFigure 9. Contributions of Input Features on Mortality Predictions for Combined Operations

This supplemental material has been provided by the authors to give readers additional information about their work.

© 2022 Castela Forte J et al. *JAMA Network Open.*

eTable 1. Grid Search Hyperparameter Ranges for Long Short-Term Memory Neural Network and Extreme Gradient-Boosting Machine Algorithms

Algorithm	Grid search parameters with intervals and final parameter values	
LSTM	Epochs	70 [50-150]
	Neurons	128 [32-128]
	Batch size	64 [16-64]
	Dropout	0.6 [0.0-0.6]
XGBoost	Number of estimators	300 [100-300]
	Learning rate	0.01 [0.01-0.1]
	Minimum child weight	1 [1-5]
	Maximum depth	7 [3-9]
	Maximum delta step	1 [0-3]
	Subsample	0.8 [0.0-1.0]
	Scale position weight	2.5 [0.3-1.0]

© 2022 Castela Forte J et al. *JAMA Network Open.*

eTable 2. Complete Demographic Characteristics and Preoperative, Intraoperative, and Postoperative Variables

Variable	No. (%) (N = 9415)			Missingness (%)
	Survivors (n = 8611)	*Non-survivors (n = 804)*	*P-value*	
**Summary pre- and post-operative and demographics variables, mean (95% CI)**				
BMI	20.4 (20.4,20.4)	20.63 (20.5,20.8)	P < 0.001	0.0
Age, median (IQR), years	67.0 (74.0)	71.0 (77.0)	P < 0.001	0.0
Sex, N (%)				
Women	2554 (27.1)	270 (2.9)	0.020	0.0
Men	6057 (64.3)	534 (5.7)		0.0
Type of operation, N (%)				0.0
CABG surgery, N (%)	5126 (59.5)	421 (52.4)	P < 0.001	
Combined surgery, N (%)	1175 (13.6)	158 (19.7)	P < 0.001	
Valve surgery, N (%)	2310 (26.8)	225 (28.0)	P < 0.001	
Preoperative creatinine, mg/dL	1.10 (1.09,1.11)	1.43 (1.35,1.52)	P < 0.001	5.3
Postoperative creatinine, mg/dL	1.02 (1.01,1.03)	1.49 (1.41,1.56)	P < 0.001	0.2
Maximum postoperative creatinine, mg/dL	108.9 (107.2,110.7)	184.5 (172.7,196.3)	P < 0.001	0.2
Preoperative urea, mg/dL	22.1 (21.6,22.4)	27.2 (25.8,28.3)	P < 0.001	7.4
Postoperative urea, mg/dL	35.6 (34.5,36.7)	42.3 (39.5,45.1)	P < 0.001	7.1
Preoperative LDH, U/L	233.3 (230.9,235.7)	287.7 (268.3,307.1)	P < 0.001	12.3
Postoperative LDH, U/L	353.4 (349.4,357.4)	623.6 (580.1,667.1)	P < 0.001	1.1
Maximum postoperative LDH, U/L	439.2 (421.5,457.0)	1216.8 (988.1,1445.8)	P < 0.001	1.1
Postoperative blood glucose, mg/dL	158.6 (158.5,158.7)	169.4 (165.8,171.2)	P < 0.001	0.3
Maximum postoperative glucose, mg/dL	205.4 (203.6,207.2)	236.4 (230.6,241.4)	P < 0.001	0.3
Preoperative Hb, g/dL	15.31 (14.99,15.47)	16.44 (14.50,18.53)	0.03	6.2
Postoperative Hb, g/dL	10.15 (9.99,10.31)	9.67 (9.35,9.99)	P < 0.001	0.2
Minimum postoperative Hb, g/dL	8.06 (8.06,8.22)	7.57 (7.41,7.73)	0.30	0.2
Preoperative thrombocytes, x10^3/μL	237.4 (235.8,238.9)	233.6 (227.5,239.8)	0.17	6.9
Postoperative thrombocytes, x10^3/μL	161.3 (160.2,162.5)	144.5 (140.1,148.9)	P < 0.001	0.4
Preoperative leukocytes, x10^3/μL	8.5 (8.4,8.7)	10.0 (8.0,12.0)	P < 0.001	7.6
Postoperative leukocytes, x10^3/μL	13.8 (13.7,13.9)	15.2 (14.3,16.0)	P < 0.001	0.7
Preoperative ALAT,U/L	36.9 (35.9,37.9)	34.9 (32.3,37.5)	0.22	12.7
Postoperative ALAT, U/L	40.6 (38.4,42.9)	103.4 (85.3,121.5)	P < 0.001	6.4

© 2022 Castela Forte J et al. *JAMA Network Open.*

Preoperative ASAT, U/L	34.3 (33.3,35.3)	41.5 (35.7,47.2)	P < 0.001	12,8
Postoperative ASAT, U/L	56.4 (54.5,58.2)	157.5 (132.9,182.0)	P < 0.001	6.2
Preoperative ESR, mm/hour	19.7 (19.3,20.0)	27.7 (26.1,29.3)	P < 0.001	26.2
Postoperative neutrophils, x10^3/µL	11.8 (11.8,11.9)	12.5 (12.1,12.8)	P < 0.001	34.8
Postoperative monocytes, x10^3/µL	3.6 (3.6,3.7)	3.7 (3.6,3.9)	0.39	30.1
Postoperative lymphocytes, x10^3/µL	1.4 (1.3,1.4)	1.9 (1.6,2.1)	0.004	30.5
Preoperative eCCR (ml/min/1.73 m^2)	76.3 (72.8,79.8)	83.2 (72.8,93.6)	0.112	96.1
Postoperative eCCR (ml/min/1.73 m^2)	92.3 (90.2,94.0)	84.9 (80.1,89.8)	P < 0.001	64.5
**Intraoperative variables**				
HR during perfusion, bpm	63.2 (62.5,64.0)	67.6 (65.3,69.9)	0.001	20.4
SBP during perfusion, mmHg	70.6 (70.4,70.9)	69.5 (68.5,70.5)	0.02	16.2
DBP during perfusion, mmHg	58.3 (58.1,58.6)	56.4 (55.5,57.3)	P < 0.001	16.2
CVP during perfusion, mmHg	6.8 (6.7,6.9)	7.6 (6.7,8.5)	0.002	11.3
CETCO2 during perfusion,	1.08 (1.07,1.09)	1.2 (1.11,1.20)	P < 0.001	9.9
HR at start surgery, bpm	68.1 (67.6,68.6)	73.4 (71.7,75.2)	P < 0.001	16.2
SBP at start surgery, mmHg	114.5 (113.4,115.6)	116.4 (112.5,120.4)	0.32	16.2
DBP at start surgery, mmHg	65.3 (64.2,66.3)	64.7 (61.0,68.4)	0.76	16.2
CVP at start surgery, mmHg	11.8 (11.0,12.5)	13.9 (11.0,16.9)	0.10	11.3
CETCO2 at start surgery	2.8 (2.7,2.8)	2.805 (2.7,2.9)	0.77	9.9
Duration of perfusion, min	127.4 (126.2,128.6)	152.7 (146.9,158.5)	P < 0.001	0.0
Maximum CPB flow	37.6 (36.6,38.5)	38.7 (35.3,42.1)	0.51	0.0
Minimum body temperature, C	28.4 (28.3,28.5)	27.5 (27.2,27.8)	P < 0.001	37.0
**Tiime-series laboratory values, mean (95% CI)**				
eCCR within 24 hours before surgery (ml/min/1.73 m^2)	64.2 (62.8,64.9)	58.9 (52.0,67.9)	0.096	83.0
Postoperative eCCR (ml/min/1.73 m^2)	92.3 (90.2,94.0)	84.9 (80.1,89.8)	P < 0.001	64.5
eCCR 0-6 hours after surgery (ml/min/1.73 m^2)	74.2 (71.8,76.7)	80.4 (67.9,92.6)	0.013	55.8
eCCR 6-12 hours after surgery (ml/min/1.73 m^2)	83.2 (80.1,86.7)	72.2 (63.8,80.5)	P < 0.001	65.5
eCCR 12-24 hours after surgery (ml/min/1.73 m^2)	76.3 (74.2,78.7)	71.45 (65.9,77.0)	0.051	60.3
eCCR at day 2 after surgery (ml/min/1.73 m^2)	84.3 (81.5,87.4)	77.3 (70.8,83.9)	0.001	72.1
eCCR at day 3 after surgery (ml/min/1.73 m^2)	83.9 (81.9,86.0)	78.38 (70.1,86.4)	P < 0.001	85.0
eCCR at day 4 after surgery (ml/min/1.73 m^2)	87.7 (86.0,89.5)	83.9 (76.3,91.2)	P < 0.001	85.4

© 2022 Castela Forte J et al. *JAMA Network Open.*

Per-operative eCCR decrease (ml/min/1.73 m^2)	14.2 (10.8,18.0)	24.3 (13.5,35.3)	0.099	83.0
Per-operative eCCR ratio	2.0 (1.9,2.1)	2.3 (1.9,2.7)	0.152	83.0
Creatinine within 24 hours before surgery, mg/dL	0.98 (0.97,1.00)	1.30 (1.22,1.37)	P < 0.001	68.0
Creatinine 0-6 hours after surgery, mg/dL	0.95 (0.94,0.96)	1.36 (1.28,1.44)	P < 0.001	66.3
Creatinine 12-24 hours after surgery, mg/dL	0.94 (0.92,0.95)	1.34 (1.26,1.42)	P < 0.001	82.3
Creatinine 24 hours after surgery, mg/dL	0.99 (0.98,1.00)	1.40 (1.32,1.48)	P < 0.001	14.9
Creatinine at day 2 after surgery, mg/dL	1.10 (1.08,1.11)	1.55 (1.47,1.63)	P < 0.001	4.3
Creatinine at day 3 after surgery, mg/dL	1.08 (1.06,1.09)	1.62 (1.52,1.71)	P < 0.001	64.2
Creatinine at day 4 after surgery, mg/dL	1.05 (1.04,1.07)	1.56 (1.47,1.66)	P < 0.001	56.4
Absolute difference in creatinine, mg/dL	15.7 (15.0,16.3)	32.5 (28.6,36.4)	P < 0.001	5.3
Relative difference in creatinine	-11.5 (-12.1,-10.8)	-2.2 (-4.8,0.4)	P < 0.001	5.3
Percentual difference in creatinine, %	22.4 (18.8,26.1)	24.6 (22.0,27.1)	0.72	5.3
Urea within 24 hours before surgery, mg/dL	35.3 (33.9,36.7)	37.3 (34.7,40.1)	0.38	69.3
Urea 0-6 hours after surgery, mg/dL	59.4 (57.9,60.5)	59.9 (56.0,63.6)	P < 0.001	66.7
Urea 6-12 hours after surgery, mg/dL	100.6 (98.3,102.8)	98.9 (92.7,105.0)	0.16	83.3
Urea 12-24 hours after surgery, mg/dL	27.2 (26.3,28.3)	31.9 (29.7,34.2)	P < 0.001	15.3
Urea at day 2 after surgery, mg/dL	38.1 (36.1,39.8)	45.9 (41.7,50.4)	P < 0.001	4.5
Urea at day 3 after surgery, mg/dL	41.7 (39.5,44.0)	50.4 (43.1,57.9)	P < 0.001	64.6
Urea at day 4 after surgery, mg/dL	27.5 (24.9,30.3)	51.5 (42.6,60.8)	P < 0.001	56.7
LDH within 24 hours before surgery, U/L	244.1 (240.8,247.4)	285.7 (261.1,310.3)	P < 0.001	75.1
LDH 0-6 hours after surgery, U/L	296.9 (293.6,300.3)	422.6 (376.1,469.1)	P < 0.001	72.4
LDH 6-12 hours after surgery, U/L	364.4 (361.4,367.4)	520.1 (487.8,552.4)	P < 0.001	76.5
LDH 12- 24 hours after surgery, U/L	370.6 (365.3,375.9)	606.1 (538.5,673.8)	P < 0.001	17.5
LDH at day 2 after surgery, U/L	370.3 (364.7,375.9)	606.3 (553.1,659.5)	P < 0.001	8.9
LDH at day 3 after surgery, U/L	408.2 (398.8,417.7)	627.1 (557.1,697.2)	P < 0.001	71.0
LDH at day 4 after surgery, U/L	366.2 (362.2,370.2)	619.9 (539.0,700.8)	P < 0.001	62.3
Blood glucose 0-6 hours after surgery, mg/dL	167.5 (167.6,169.4)	185.5 (181.9,189.1)	P < 0.001	8.8
Blood glucose 6-12 hours after surgery, mg/dL	176.6 (174.7,178.4)	194.6 (189.1,196.4)	P < 0.001	13.6
Blood glucose 12-24 hours after surgery, mg/dL	153.2 (151.4,153.2)	169.4 (165.2,171.2)	P < 0.001	8.2

© 2022 Castela Forte J et al. *JAMA Network Open.*

Blood glucose at day 2 after surgery, mg/dL	153.2 (151.3,153.1)	163.9 (160.4,167.6)	P < 0.001	65.7
Blood glucose at day 3 after surgery, mg/dL	163.9 (162.2,163.9)	173.0 (167.6,176.6)	0.003	78.4
Blood glucose at day 4 after surgery, mg/dL	158.6 (156.8,158.6)	160.4 (156.8,163.9)	0.16	83.6
Hb within 24 hours before surgery, g/dL	14.66 (14.34,15.15)	16.62 (14.02,19.17)	0.01	64.0
Hb 0-6 hours after surgery, g/dL	9.35 (9.18,9.35)	9.02 (8.86,9.02)	P < 0.001	4.3
Hb 6-12 hours after surgery, g/dL	9.83 (9.67,9.99)	9.35 (9.35,9.51)	P < 0.001	7.3
Hb 12-24 hours after surgery, g/dL	9.99 (9.83,9.99)	9.51 (9.51,9.67)	P < 0.001	5.3
Hb at day 2 after surgery, g/dL	10.63 (10.15,11.12)	9.51 (9.35,9.67)	P < 0.001	4.0
Hb at day 3 after surgery, g/dL	9.83 (9.67,10.15)	9.67 (9.35,10.15)	P < 0.001	63.0
Hb at day 4 after surgery, g/dL	10.15 (9.83,10.47)	10.15 (8.86,11.28)	P < 0.001	55.5
Leukocytes within 24 hours before surgery, x10^3/μL	8.3 (8.1,8.5)	10.5 (6.9,14.1)	0.001	69.3
Leukocytes 0-6 hours after surgery, x10^3/μL	13.4 (13.3,13.5)	14.3 (13.8,14.8)	0.001	55.5
Leukocytes 6-12 hours after surgery, x10^3/μL	12.8 (12.8,12.9)	12.7 (12.4,13.0)	0.09	80.2
Leukocytes 12-24 hours after surgery, x10^3/μL	13.9 (13.8,14.0)	13.8 (13.4,14.1)	0.06	15.7
Leukocytes at day 2 after surgery, x10^3/μL	16.7 (16.5,16.7)	17.0 (16.6,17.4)	0.08	6.1
Leukocytes at day 3 after surgery, x10^3/μL	14.7 (14.6,14.9)	15.7 (14.9,15.6)	P < 0.001	67.1
Leukocytes at day 4 after surgery, x10^3/μL	12.3 (11.9,12.7)	15.4 (11.9,18.9)	P < 0.001	59.4
Thrombocytes within 24 hours before surgery, x10^3/μL	224.7 (223.1,226.4)	220.0 (213.3,226.6)	0.11	66.1
Thrombocytes 0-6 hours after surgery, x10^3/μL	149.9 (148.8,151.0)	143.6 (139.3,147.9)	P < 0.001	7.6
Thrombocytes 6-12 hours after surgery, x10^3/μL	162.1 (160.9,163.3)	145.6 (141.2,149.9)	P < 0.001	35.6
Thrombocytes 12-24 hours after surgery, x10^3/μL	164.3 (163.1,165.6)	145.7 (141.3,150.1)	P < 0.001	12.2
Thrombocytes at day 2 after surgery, x10^3/μL	158.6 (157.4,159.7)	139.2 (134.9,143.5)	P < 0.001	55.0
Thrombocytes at day 3 after surgery, x10^3/μL	153.4 (152.3,154.6)	133.1 (128.6,137.7)	P < 0.001	78.2
Thrombocytes at day 4 after surgery, x10^3/μL	168.5 (167.1,169.8)	141.9 (136.4,147.4)	P < 0.001	74.8
ALAT within 24 hours before surgery, U/L	41.2 (40.2,42.2)	43.4 (40.0,46.8)	0.21	74.8
ALAT 0-6 hours after surgery, U/L	32.2 (31.4,32.9)	37.9 (32.3,43.6)	0.07	61.9
ALAT 6-12 hours after surgery, U/L	38.4 (37.7,39.1)	51.4 (43.4,59.5)	P < 0.001	78.7
ALAT 12-24 hours after surgery, U/L	35.1 (32.8,37.4)	61.5 (44.2,78.9)	0.11	20.9
ALAT at day 2 after surgery, U/L	37.1 (34.5,39.7)	74.9 (55.5,94.2)	P < 0.001	43.1

© 2022 Castela Forte J et al. *JAMA Network Open.*

ALAT at day 3 after surgery, U/L	63.6 (61.0,66.2)	110.2 (86.2,134.3)	0.04	77.5
ALAT at day 4 after surgery, U/L	58.3 (55.8,60.7)	115.5 (85.8,145.2)	0.31	70.4
ASAT within 24 hours before surgery, U/L	44.8 (43.1,46.4)	49.7 (40.4,58.9)	0.11	74.7
ASAT 0-6 hours after surgery, U/L	49.4 (47.7,51.1)	77.8 (62.1,93.5)	0.66	64.1
ASAT 6-12 hours after surgery, U/L	74.5 (72.9,76.1)	119.1 (104.9,133.3)	P < 0.001	78.7
ASAT 12-24 hours after surgery, U/L	70.2 (67.6,72.7)	157.6 (123.1,192.2)	P < 0.001	21.1
ASAT at day 2 after surgery, U/L	60.2 (57.2,63.1)	148.8 (112.0,185.6)	P < 0.001	12.6
ASAT at day 3 after surgery, U/L	101.2 (98.3,104.0)	142.8 (113.8,171.8)	0.002	72.2
ASAT at day 4 after surgery, U/L	67.6 (65.2,70.1)	158.8 (115.2,202.5)	0.11	63.5
ESR within 24 hours before surgery, mm/hour	19.5 (19.1,19.8)	27.8 (26.2,29.5)	P < 0.001	83.3
Neutrophils 0-6 hours after surgery, x10^3/µL	11.3 (11.1,11.2)	11.3 (10.9,11.7)	0.96	98.9
Neutrophils 6-12 hours after surgery, x10^3/µL	11.1 (11.1,11.2)	10.6 (10.3,11.0)	0.001	94.2
Neutrophils 12-24 hours after surgery, x10^3/µL	11.8 (11.7,11.9)	11.4 (11.0,11.7)	0.002	44.5
Neutrophils at day 2 after surgery, x10^3/µL	13.7 (13.6,13.8)	13.9 (13.6,14.3)	0.18	88.2
Neutrophils at day 3 after surgery, x10^3/µL	11.8 (11.7,11.9)	12.5 (12.2,12.8)	P < 0.001	92.4
Neutrophils at day 4 after surgery, x10^3/µL	9.6 (9.3,9.9)	11.9 (9.3,14.5)	P < 0.001	94.2
Monocytes 0-6 hours after surgery, x10^3/µL	2.9 (2.8,2.9)	2.5 (2.1,2.9)	0.81	98.7
Monocytes 6-12 hours after surgery, x10^3/µL	3.2 (3.1,3.2)	3.3 (3.1,3.4)	0.22	93.8
Monocytes 12-24 hours after surgery, x10^3/µL	3.4 (3.4,3.5)	3.4 (3.3,3.6)	0.74	39.4
Monocytes at day 2 after surgery, x10^3/µL	4.2 (4.2,4.2)	4.3 (4.1,4.5)	0.67	85.3
Monocytes at day 3 after surgery, x10^3/µL	3.9 (3.8,3.9)	3.8 (3.7,4.0)	0.03	90.8
Monocytes at day 4 after surgery, x10^3/µL	3.8 (3.8,3.9)	3.9 (3.7,4.0)	0.05	92.8
Lymphocytes 0-6 hours after surgery, x10^3/µL	1.5 (1.4,1.5)	2.3 (2.0,2.6)	P < 0.001	98.7
Lymphocytes 6-12 hours after surgery, x10^3/µL	0.9 (0.8,0.9)	1.3 (1.0,1.6)	0.09	93.8
Lymphocytes 12-24 hours after surgery, x10^3/µL	1.2 (1.1,1.2)	1.4 (1.2,1.6)	0.86	39.5
Lymphocytes at day 2 after surgery, x10^3/µL	1.5 (1.5,1.6)	1.6 (1.4,1.8)	P < 0.001	85.3
Lymphocytes at day 3 after surgery, x10^3/µL	1.9 (1.8,1.9)	2.0 (1.8,2.2)	P < 0.001	90.9
Lymphocytes at day 4 after surgery, x10^3/µL	2.2 (2.1,2.2)	2.6 (2.3,2.9)	0.56	92.8
**Euroscore**				47.3
Euroscore I	5.8 (5.7,5.9)	8.9 (8.4,9.4)	P < 0.001	

© 2022 Castela Forte J et al. *JAMA Network Open.*

Creatinine value, µmol/ L	95.8 (92.1,99.4)	130.4 (105.8,154.9)	P < 0.001	
Chronic pulmonary disease	735 (15.9)	86 (27.2)	P < 0.001	
Extracardiac arteriopathy	926 (20.1)	95 (30.4)	P < 0.001	
Neurological dysfunction	249 (5.4)	34 (10.8)	P < 0.001	
Previous Cardiac Surgery	220 (4.8)	24 (7.6)	0.04	
Creatinine > 200 µmol/ L	218 (8.1)	53 (23.9)	P < 0.001	
Active endocarditis	53 (1.2)	17 (5.4)	P < 0.001	
Critical preoperative state	305 (6.7)	63 (20.0)	P < 0.001	
Unstable angina	871 (19.0)	82 (26.1)	P < 0.001	
LV function				
Good	2930 (36.5)	154 (20.6)	P < 0.001	
Moderate	1360 (16.9)	111 (14.8)	0.15	
Poor	292 (3.6)	49 (6.5)	P < 0.001	
Recent MI	785 (17.0)	57 (18.0)	0.71	
Pulmonary hypertension	134 (2.9)	29 (9.2)	P < 0.001	
Emergency	210 (7.8)	38 (17.2)	P < 0.001	
Other than isolated CABG	1747 (37.9)	152 (48.1)	P < 0.001	
Surgery on thoracic aorta	140 (3.0)	111 (3.5)	0.77	
Post infarct septal rupture	6 (0.13)	1 (0.32)	0.94	
Urgency				
Elective	1710 (21.3)	71 (9.5)	P < 0.001	
Life-threatening	34 (0.42)	8 (1.07)	0.03	
Urgent	168 (2.1)	16 (2.1)	0.96	
**Acute kidney injury, N (%)**			<0.001	15.3%
AKI 0	6398 (93.9)	418 (6.1)	<0.001	
AKI 1	712 (81.8)	159 (18.2)	<0.001	
AKI 2	67 (61.5)	42 (38.5)	<0.001	
AKI 3	98 (53.9)	84 (46.1)	<0.001	

Parametric continuous variables are presented as mean (95% CI) and compared using Student's t-test; non- parametric variables are reported as median [IQR] and compared using the Wilcoxon signed rank test. Categorical variables are represented as number, with the percentage in parentheses. BMI = body mass index, eCCR = estimated creatinine clearance, CPB = cardio-pulmonary bypass, HR = heart rate, SBP = systolic blood pressure, DBP = diastolic blood pressure, CVP = central venous pressure, PaCO2 = arterial CO2 pressure, CETCO2 = end-tidal carbon dioxide, PaO2 = arterial oxygen pressure, SaO2 = oxygen saturation, ESR = erythrocyte sedimentation rate, LDH = lactate dehydrogenase, Hb = hemoglobin, ALAT = alanine aminotransferase, ASAT = aspartate aminotransferase, LV = left ventricle, MI = myocardial infarction, CABG = coronary artery bypass grafting.

© 2022 Castela Forte J et al. *JAMA Network Open.*

eTable 3. Area Under Receiver Operating Characteristic Curves for Models for 30-d Mortality Prediction

AUROCs are presented with 95% confidence interval.

Model	AUROC
Entire perioperative period	0.81 (0.78,0.86)
Postoperative only	0.78 (0.74,0.83)
Intra- and postoperative	0.77 (0.72,0.82)
Pre- and postoperative	0.75 (0.70,0.80)
Pre- and intraoperative	0.47 (0.41,0.53)
Intraoperative only	0.58 (0.51,0.64)
Preoperative only	0.70 (0.61,0.71)

© 2022 Castela Forte J et al. *JAMA Network Open.*

eTable 4. Area Under Receiver Operating Characteristic Curves for Models for 1-y Mortality Prediction

AUROCs are presented with 95% confidence interval.

Model	AUROC
Entire perioperative period	0.82 (0.78,0.86)
Postoperative only	0.79 (0.74,0.83)
Intra- and postoperative	0.79 (0.74,0.84)
Pre- and postoperative	0.79 (0.74,0.83)
Pre- and intraoperative	0.44 (0.37,0.49)
Intraoperative only	0.51 (0.45,0.56)
Preoperative only	0.66 (0.61,0.71)

© 2022 Castela Forte J et al. *JAMA Network Open.*

eTable 5. Area Under Receiver Operating Characteristic Curves for Models for 5-y Mortality Prediction

AUROCs are presented with 95% confidence interval.

Model	AUROC
Entire perioperative period	0.79 (0.75,0.84)
Postoperative only	0.77 (0.73,0.82)
Intra- and postoperative	0.78 (0.73,0.83)
Pre- and postoperative	0.76 (0.72,0.82)
Pre- and intraoperative	0.48 (0.42,0.54)
Intraoperative only	0.51 (0.45,0.56)
Preoperative only	0.69 (0.64,0.74)

© 2022 Castela Forte J et al. *JAMA Network Open.*

eFigure 1. Survival Curves and Survival Statistics for Entire Cohort and by Operation Type. Panels A-D: Full cohort, CABG, Valve, Combined.

(A) 30-day, 1-year and 5-year survival curves for whole cohort

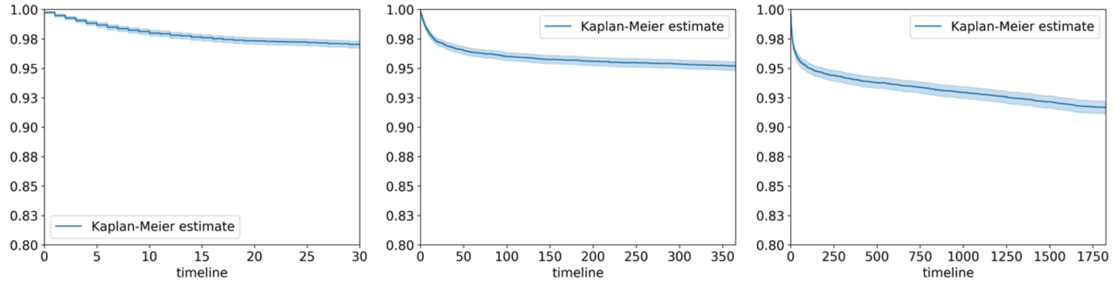

Operation type	30-day mortality	1-year mortality	5-year mortality
All	268 (3.1%)	420 (4.8%)	612 (7.0%)

(B) 30-day, 1-year and 5-year survival curves for CABG

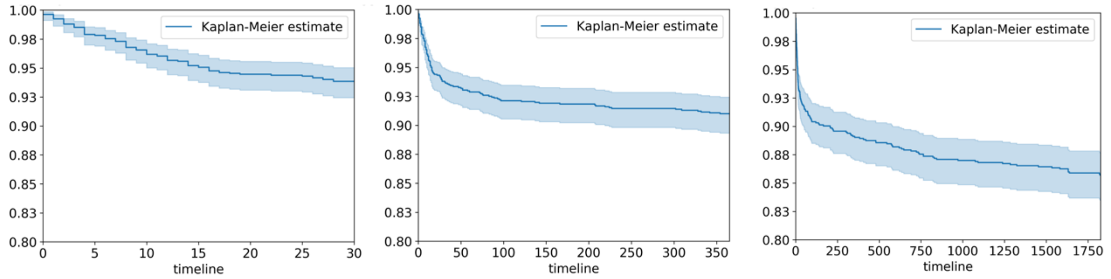

Operation type	30-day mortality	1-year mortality	5-year mortality
CABG	126 (2.4%)	191 (3.6%)	310 (5.9%)

(C) 30-day, 1-year and 5-year survival curves for valve surgery

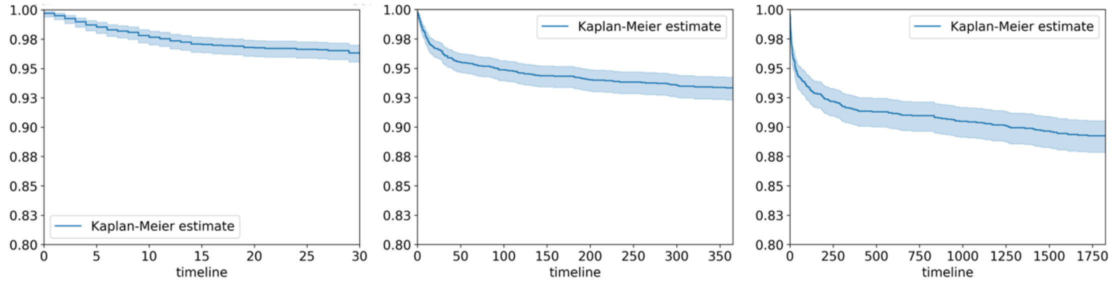

Operation type	30-day mortality	1-year mortality	5-year mortality
Valve	73 (3.2%)	132 (5.8%)	173 (7.5%)

(D) 30-day, 1-year and 5-year survival curves for combined valve and CABG surgery

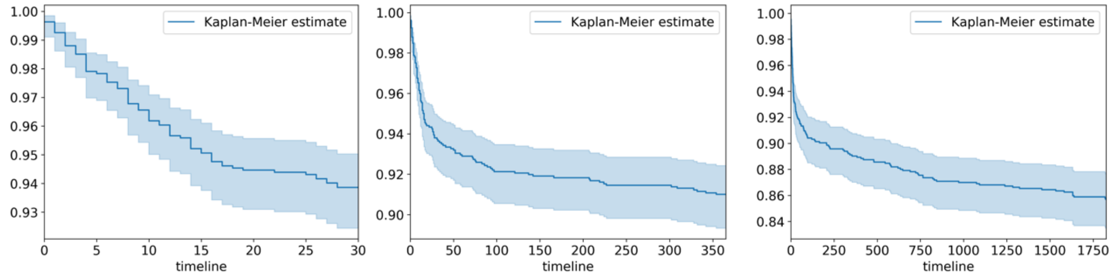

Operation type	30-day mortality	1-year mortality	5-year mortality
Combined	69 (5.7%)	97 (8.0%)	129 (10.6%)

© 2022 Castela Forte J et al. *JAMA Network Open.*

eFigure 2. Calibration Plot for Model Predicting 30-d Mortality in Full Cohort (A) entire perioperative period model and (B) postoperative period model.

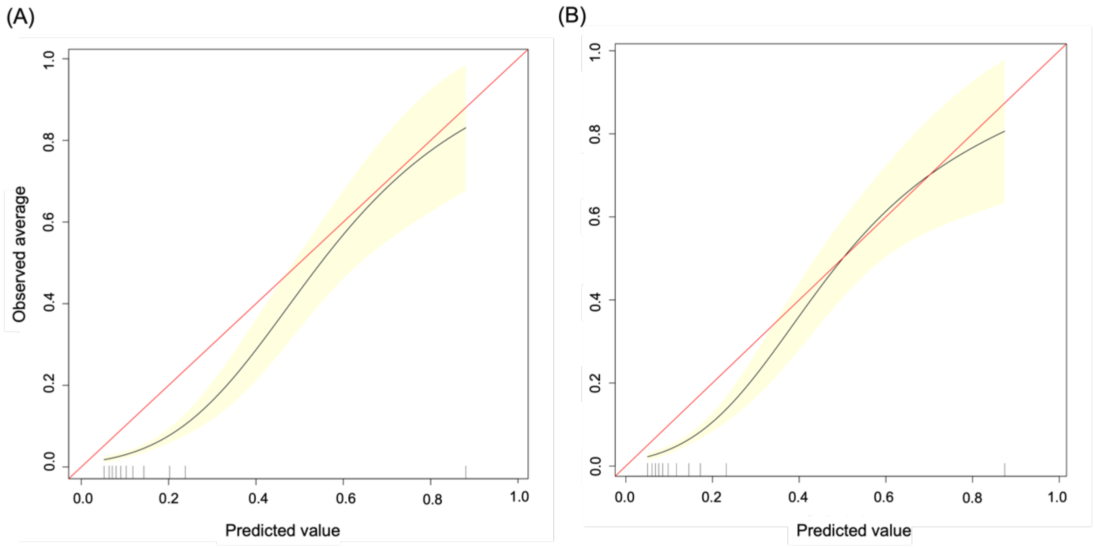

© 2022 Castela Forte J et al. *JAMA Network Open.*

eFigure 3. Calibration Plot for Model Predicting 1-y Mortality in Full Cohort (A) entire perioperative period model and (B) postoperative period model.

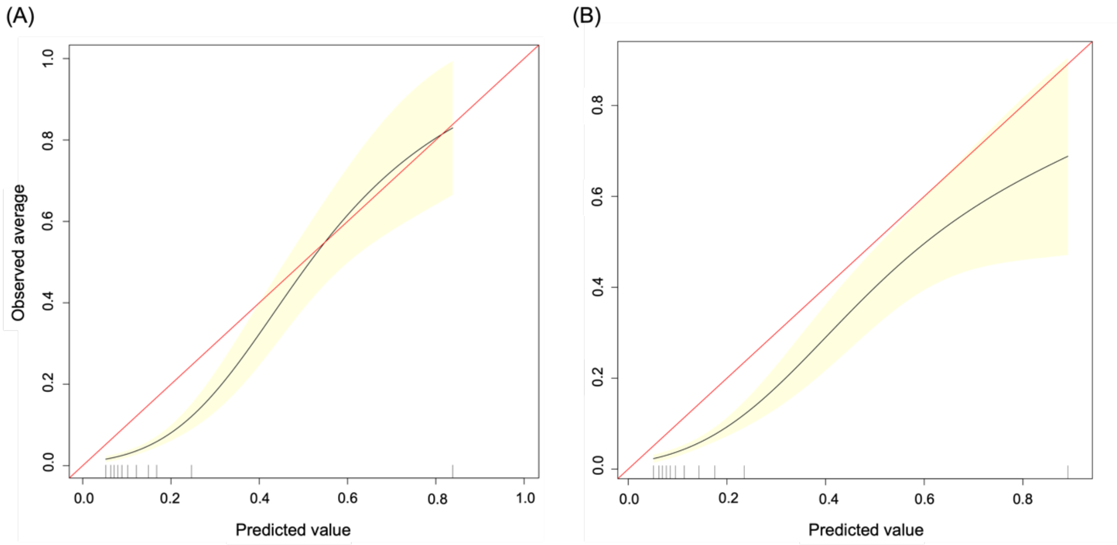

© 2022 Castela Forte J et al. *JAMA Network Open.*

eFigure 4. Calibration Plot for Model Predicting 5-y Mortality in Full Cohort (A) entire perioperative period model and (B) postoperative period model.

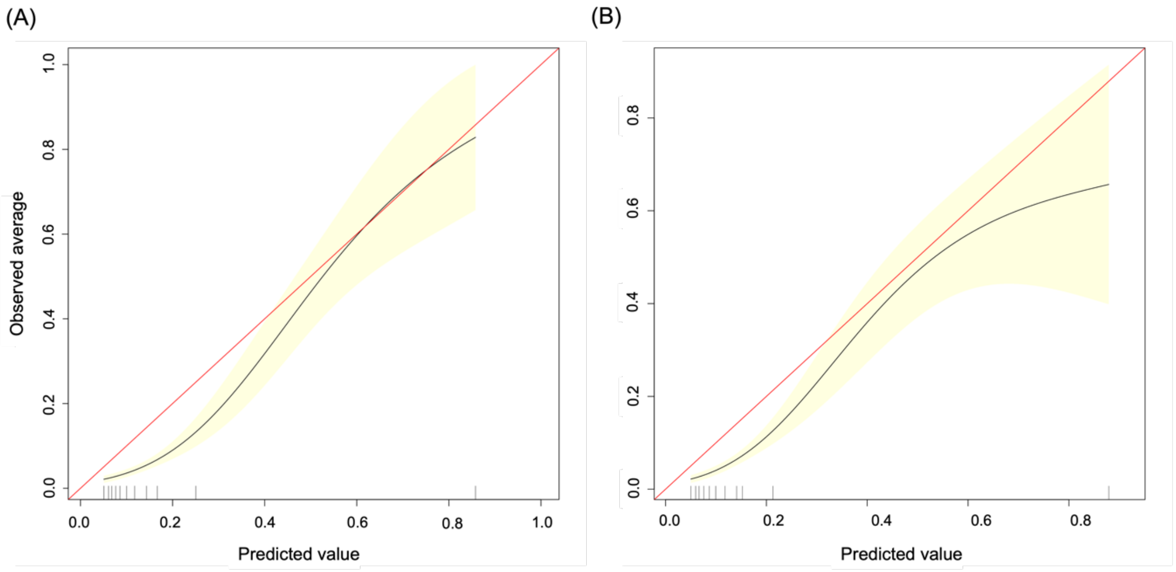

© 2022 Castela Forte J et al. *JAMA Network Open.*

eFigure 5. Predictiveness Plots for Models Including Data From All 3 Perioperative Periods
30-day mortality (A), 1-year mortality (B), and 5-year mortality (C).

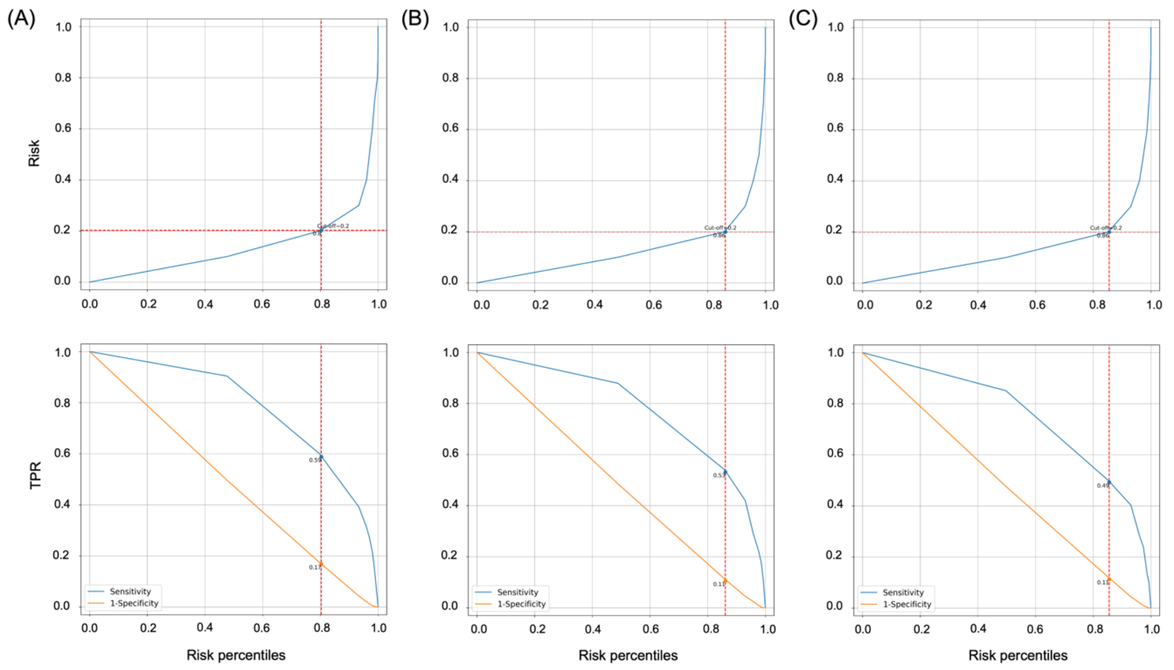

TPR = true positive rate.

© 2022 Castela Forte J et al. *JAMA Network Open.*

eFigure 6. Predictiveness Plots for Models Including Postoperative Data Only 30-day mortality (A), 1-year mortality (B), and 5-year mortality (C).

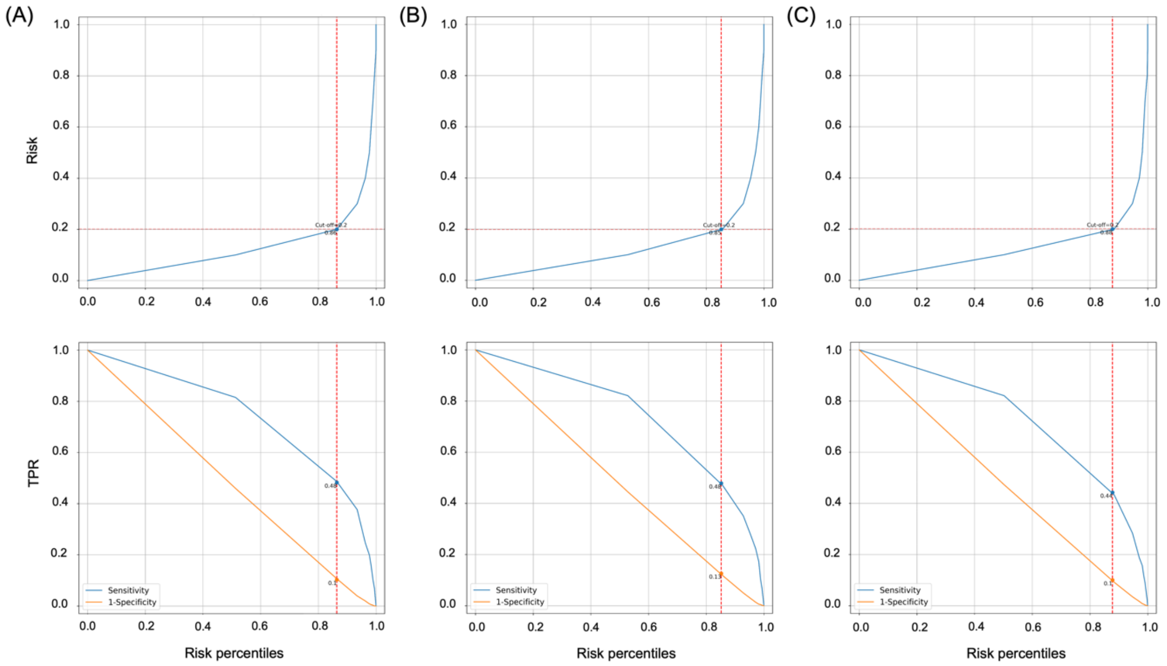

TPR = true positive rate

© 2022 Castela Forte J et al. *JAMA Network Open.*

eFigure 7. Contribution of Input Features to Mortality Predictions for Coronary Artery Bypass Grafting Operations

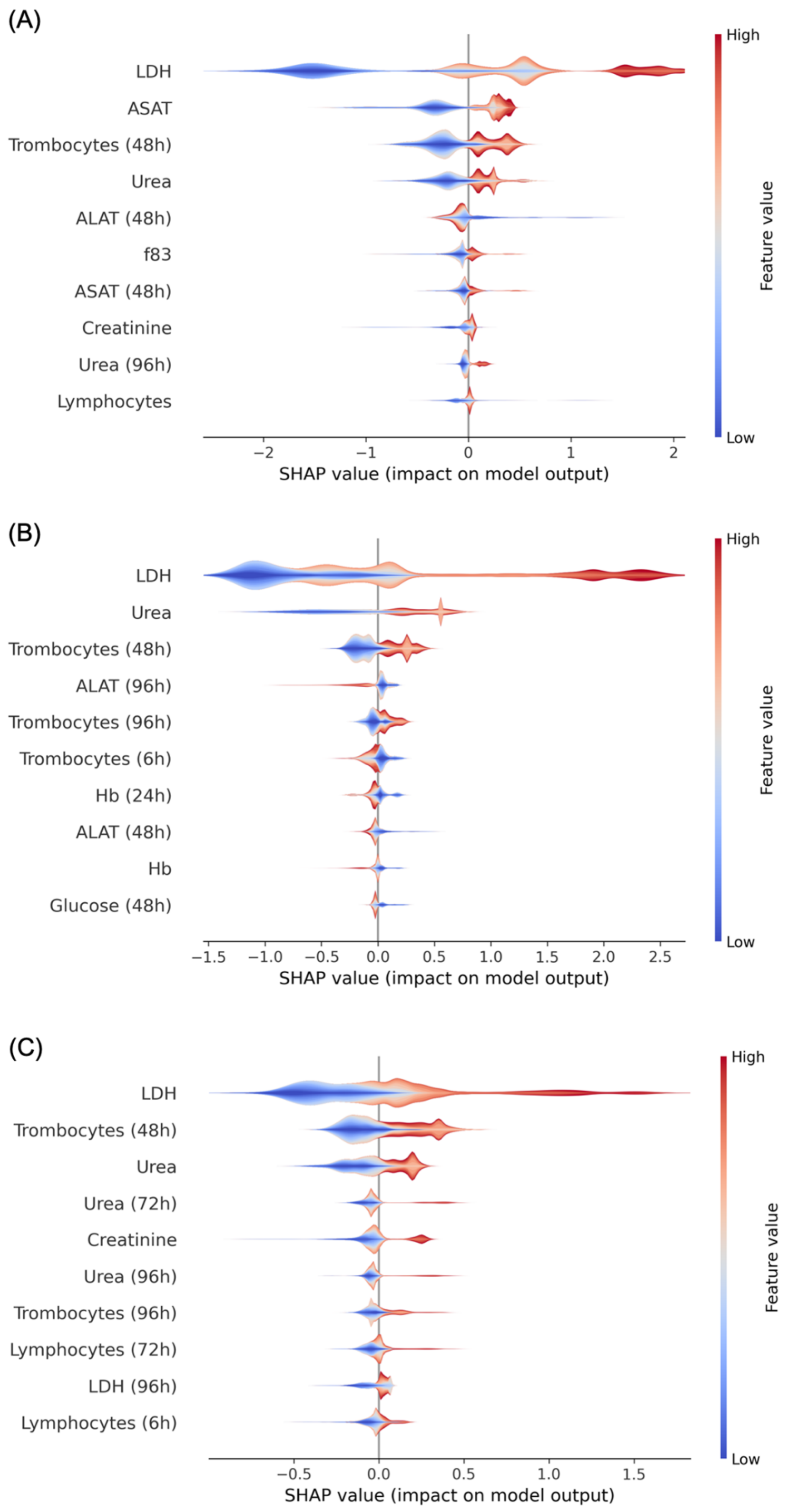

Positive SHAP values represent the theoretical impact of the feature towards mortality, with higher values represented in red, lower values in blue, and values left of the x axis driving predictions towards survival, and on the right towards mortality. (A) Top 10 features for 30-day mortality predictions. (B) Top 10 features for 1-year mortality predictions. (C) Top 10 features for 5-year mortality predictions.

© 2022 Castela Forte J et al. *JAMA Network Open.*

eFigure 8. Contributions of Input Features to Mortality Predictions for Valve Operations

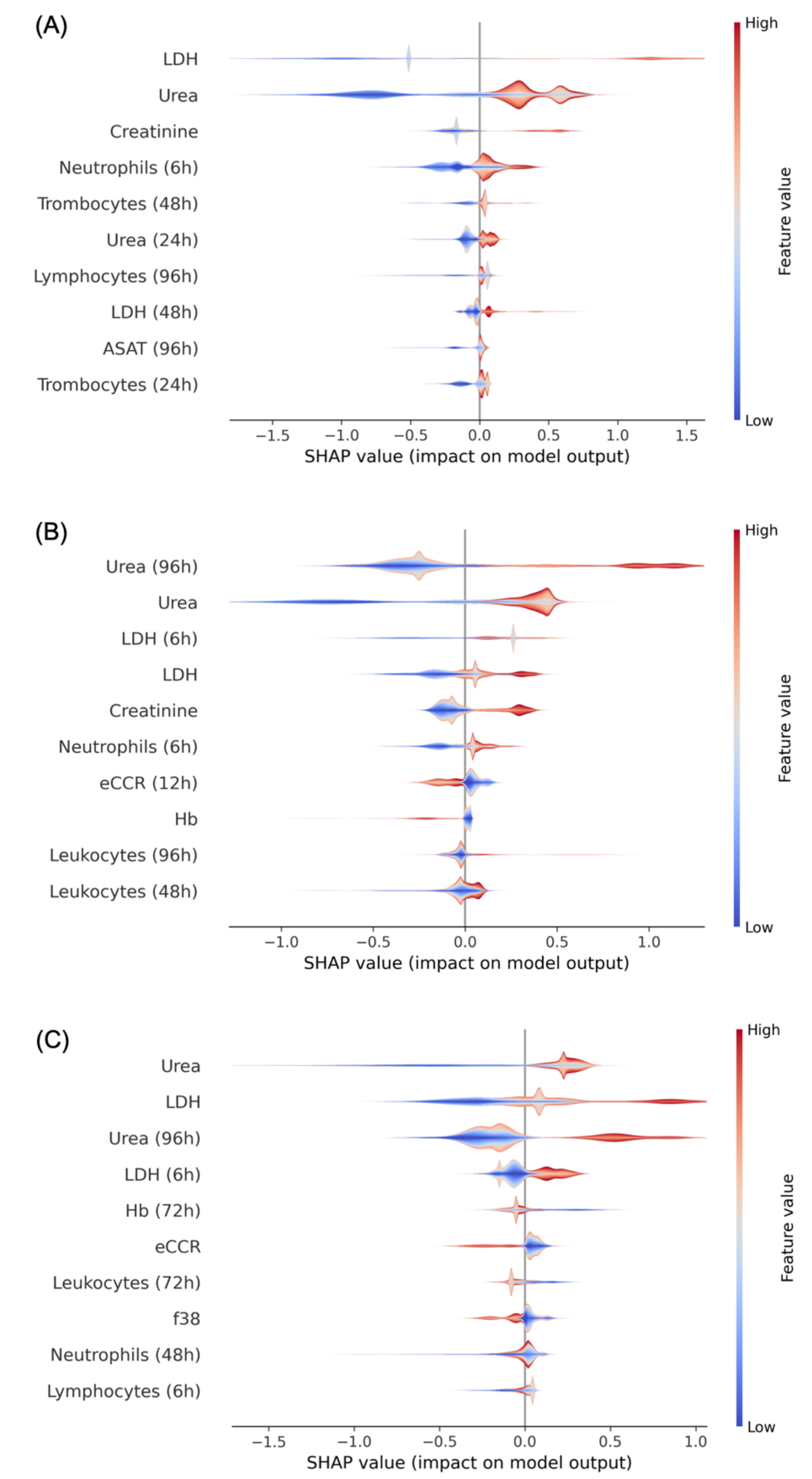

Positive SHAP values represent the theoretical impact of the feature towards mortality, with higher values represented in red, lower values in blue, and values left of the x axis driving predictions towards survival, and on the right towards mortality. (A) Top 10 features for 30-day mortality predictions. (B) Top 10 features for 1-year mortality predictions. (C) Top 10 features for 5-year mortality predictions.

© 2022 Castela Forte J et al. *JAMA Network Open.*

eFigure 9. Contributions of Input Features on Mortality Predictions for Combined Operations

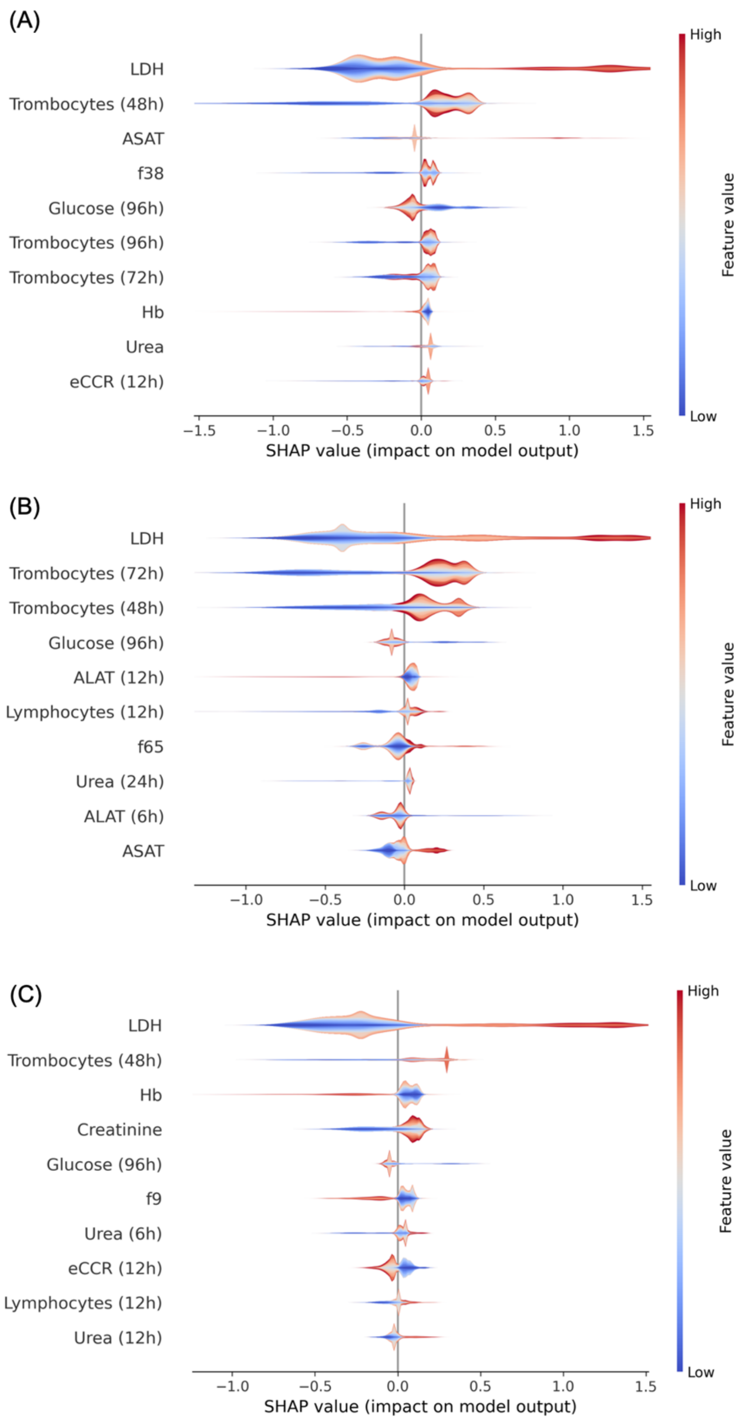

Positive SHAP values represent the theoretical impact of the feature towards mortality, with higher values represented in red, lower values in blue, and values left of the x axis driving predictions towards survival, and on the right towards mortality. (A) Top 10 features for 30-day mortality predictions. (B) Top 10 features for 1-year mortality predictions. (C) Top 10 features for 5-year mortality predictions.

© 2022 Castela Forte J et al. *JAMA Network Open.*
